# Balancing anti-inflammatory and anti-oxidant responses in murine bone marrow derived macrophages

**DOI:** 10.1371/journal.pone.0184469

**Published:** 2017-09-08

**Authors:** Christopher R. Nitkin, Tracey L. Bonfield

**Affiliations:** 1 Department of Pediatrics, Division of Neonatology, Rainbow Babies and Children’s Hospital, Cleveland, Ohio, United States of America; 2 Department of Pediatrics, Division of Pediatric Pulmonology, Case Western Reserve University, Cleveland, Ohio, United States of America; National Institutes of Health, UNITED STATES

## Abstract

**Rationale:**

The underlying pathophysiology of bronchopulmonary dysplasia includes a macrophage-mediated host response orchestrated by anti-inflammatory peroxisome proliferator-activated receptor gamma (PPARγ) and anti-oxidant nuclear factor (erythroid-derived 2)-like 2 (Nrf2). These have not yet been studied in combination. This study tested the hypothesis that combined inflammatory and oxidative stressors would interact and change PPARγ- and Nrf2-regulated gene expression and antioxidant capacity. Therefore, we investigated the effect of dual stimulation with lipopolysaccharide and hyperoxia in murine bone marrow-derived macrophages (BMDM).

**Methods:**

Sub-confluent BMDM from wild-type C57BL/6J mice were treated with lipopolysaccharide (LPS) 1ug/mL for 2 hours followed by room air (21% oxygen) or hyperoxia (95% oxygen) for 24 hours. Taqman real time-polymerase chain reaction gene expression assays, total antioxidant capacity assays, and Luminex assays were performed.

**Results:**

Supernatants of cultured BMDM contained significant antioxidant capacity. In room air, LPS treatment decreased expression of PPARγ and Nrf2, and increased expression of tumor necrosis factor-alpha and heme oxygenase-1; similar findings were observed under hyperoxic conditions. LPS treatment decreased cellular total antioxidant capacity in room air but not in hyperoxia. Increased expression of sulfiredoxin-1 in response to hyperoxia was not observed in LPS-treated cells. Dual stimulation with LPS treatment and exposure to hyperoxia did not have synergistic effects on gene expression. Cellular total antioxidant capacity was not changed by hyperoxia exposure.

**Conclusions:**

Our hypothesis was supported and we demonstrate an interaction between inflammatory and oxidative stressors in a model system of bronchopulmonary dysplasia pathogenesis. The protective anti-oxidant effect of cell culture media may have protected the cells from the most deleterious effects of hyperoxia.

## Introduction

Bronchopulmonary dysplasia (BPD) is the major pulmonary morbidity of preterm birth, affecting up to 45% of very low birth-weight infants (less than 1500 grams) [1]. As many as 10% of these infants die and many experience neurodevelopmental and cognitive impairment [2]. A diagnosis of BPD more than doubles the cost of initial hospitalization [3] and places significant financial and emotional stresses on the family. Advances in neonatal care such as gentle ventilation, judicious use of steroids, caffeine, vitamin A, and appropriate nutrition have improved many outcomes in premature infants [4]. However, there is currently no cure for BPD, and two of the most significant pathogenic factors, infection and supplemental oxygen therapy [5], are often unavoidable.

The underlying pathophysiology of BPD is multifactorial but includes inflammation and oxidative stress which lead to airway remodeling, alveolar simplification, and pulmonary vasculature abnormalities [5,6]. BPD research has historically focused on inflammation, but the antioxidant response is also a significant contributor [7]. Our study sought to understand the roles of both inflammation and oxidative stress, including the two transcription factors peroxisome proliferator-activated receptor gamma (PPARγ) [8] and nuclear factor (erythroid-derived 2)-like 2 (Nrf2) [9], which coordinate the anti-inflammatory and anti-oxidant responses, respectively, as well as the downstream effectors tumor necrosis factor-α (TNFα) (pro-inflammatory), sulfiredoxin (Srxn1) (anti-oxidant), and heme oxygenase-1 (Hmox1) (anti-inflammatory and anti-oxidant). To investigate the molecular pathways responsible for the cellular response to the pathogenic stimuli important in BPD, we have developed an *in vitro* model of BPD using murine bone marrow-derived macrophages (BMDM) treated with lipopolysaccharide (LPS), an inflammatory stimulus, and hyperoxia, an oxidative stress. This study tested the hypothesis that combined inflammatory and oxidative stressors would interact and change PPARγ- and Nrf2-regulated gene expression and antioxidant capacity. We present data which suggests an interaction between the molecular pathways responsive to inflammatory and oxidative stressors.

## Materials and methods

### Animals

**Fig 1:**
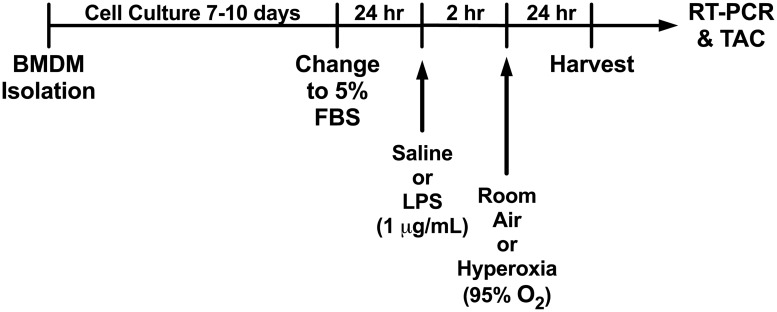
Experimental design. BMDM were isolated from wild-type C57BL/6J mice and grown to subconfluence, then were treated with LPS or saline, followed 2 hours later by hyperoxia or room air, and were harvested 24 hours later.

The experimental design is outlined in Fig 1. All studies were submitted to and approved by the Case Western Reserve University Institutional Animal Care and Use Committee (protocol 2014–0093). No surgeries were performed on living animals, and BMDM were isolated from mice euthanized with carbon dioxide and cervical dislocation.

### Cell culture

#### BMDM cell culture

BMDM were generated from bone marrow from tibia and femur of six-week-old, wild-type C57BL/6J mice as previously described [10–12]. Briefly, bone marrow was collected and plated in six-well tissue culture plates (Corning, Corning, New York, United States). BMDM were cultured in RPMI 1640-based media (Fisher Scientific, Suwanee, Georgia, United States) containing 10% heat-inactivated fetal bovine serum (Life Technologies, Waltham, Massachusetts, United States), 1% Penicillin-Streptomycin-Glutamine (Life Technologies), and 20% spent L929-cell conditioned media as a source of M-CSF [10], exchanging media every 48–72 hours. This standard cell culture media was exchanged for an equal volume of media supplemented with 5% instead of 10% FBS twenty-four hours prior to LPS/hyperoxia exposure. Following LPS/hyperoxia treatment, cells were manually removed by scraping, the suspension was centrifuged at 400 x *g* for 9 minutes, and cell pellet and supernatant were stored separately at -80°C.

#### Hyperoxia treatment

Cell culture plates were placed in a 5.7 L modular incubator chamber (Billups-Rothenberg, Del Mar, California, United States) which was then flushed with a gas mixture of 95% oxygen and 5% carbon dioxide (Airgas Corporation, Radnor Township, Pennsylvania, United States) until oxygen saturation reached 95±2% as measured with a MaxO2+AE oxygen analyzer (Maxtec, Salt Lake City, Utah, United States). This prepared chamber was placed within a standard cell culture incubator at 37°C. Control cells were kept in a standard cell culture incubator with room air (21% oxygen) supplemented with 5% carbon dioxide. Of note, hyperoxia treatment, with or without LPS treatment, did not cause any cell death or observable cellular stress, as observed from the microscopic evaluation of cell confluence.

#### LPS treatment

Aliquots of LPS (Sigma-Aldrich, St. Louis, Missouri, United States) were stored at -80°C and diluted with phosphate buffered saline (Genesee Scientific, San Diego, California, United States), then were applied to cells for a final concentration of 1 μg/mL. Control cells were treated with equal volumes of phosphate buffered saline. Of note, LPS treatment, with or without hyperoxia treatment, did not cause any cell death or observable cellular stress, as observed from the microscopic evaluation of cell confluence.

### Gene expression

RNA was isolated from cells with Ribozol reagent (Amresco, Solon, Ohio, United States) and cDNA was synthesized using qScript cDNA Synthesis Kits (VWR, Radnor, Pennsylvania, United States) and stored at -80°C. RNA and cDNA concentrations were determined by spectrophotometry (SpectraMax i3x, Molecular Devices, Sunnyvale, California, United States). cDNA was diluted in sterile nuclease-free water to which TaqMan Universal PCR Master Mix and PCR primers (Life Technologies) were added. Pre-validated TaqMan primers spanning exon junctions were used to prevent detection of genomic DNA: assay ID number PPARγ (Mm01184322_m1), TNFα (Mm00443258_m1), Nrf2 (Mm00477784_m1), Srxn1 (Mm00769566_m1), and Hmox1 (Mm00516005_m1). Samples were plated in a 96-well reaction plate, run on an Applied BioSystems 7300 Real Time PCR System, and analyzed using ABI 7300 Prism software and Microsoft Excel.

### Luminex ELISA

Cytokine levels from cell culture supernatants were assessed by Luminex assay (R&D Systems, Minneapolis, Minnesota) per manufacturer’s instructions. Supernatants were centrifuged and stored at -80°C until evaluation for TNFα. Undiluted samples were then run on a Luminex Magpix according to the manufacturer’s protocol.

### Total antioxidant capacity

Concentration of small molecule and protein antioxidants was measured using a Total Antioxidant Capacity Assay Kit (Sigma-Aldrich) per the manufacturer’s instructions. Absorbance was measured by spectrophotometry (SpectraMax i3x). Cell culture media constituents (FBS, L929-cell conditioned media, RPMI media) were assayed individually, cell culture media without cells (RPMI supplemented with 5% FBS, 10% L929-cell conditioned media) treated with LPS or saline and/or 95% hyperoxia or room air was assayed, and supernatants from harvested BMDM which had been treated with LPS or saline and/or 95% hyperoxia or room air were assayed.

### Statistical analyses

Gene expression data were normalized to GAPDH to obtain ΔCt values. Change in expression, relative to normoxia or saline controls as noted in the text, was calculated using the 2^-ΔΔCt^ method [13]. Because Gaussian distribution could not be assumed, data were analyzed with Kruskal-Wallis test with Dunn’s correction for multiple comparisons using GraphPad Prism 7.0c for Mac (GraphPad Software, La Jolla California USA). A P value less than 0.05 was considered statistically significant.

## Results

### Cell culture media has antioxidant activity

Because preliminary experiments did not reveal any significant effect of hyperoxia on gene expression, we hypothesized that the cell culture media might have antioxidant activity, protecting the cells from experiencing marked oxidative stress. Therefore, we measured total antioxidant capacity (TAC) of the cell-free culture media treated with LPS and hyperoxia, as well as FBS, L929-CM, and RPMI, plus supernatants from harvested BMDM.

**Fig 2:**
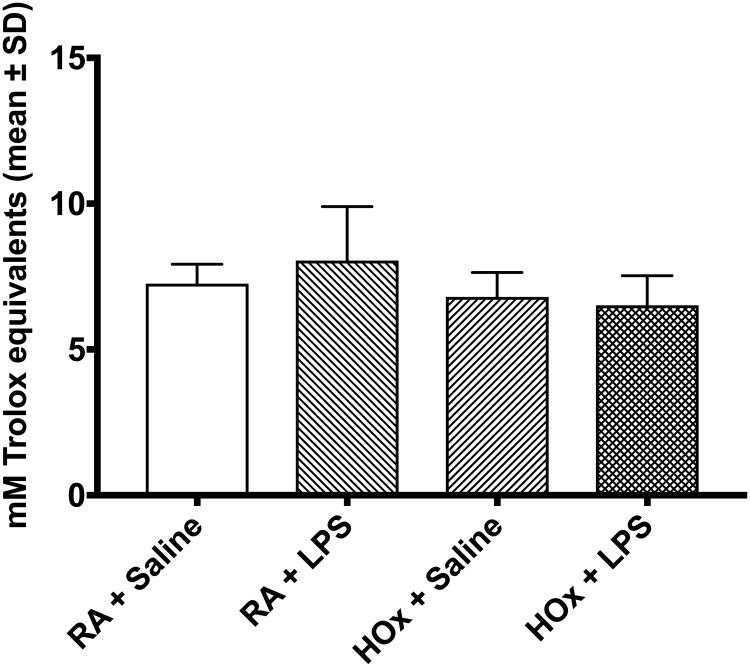
Total antioxidant capacity of BMDM supernatant. Supernatant from BMDM treated with LPS or saline and hyperoxia (HOx) or room air (RA) was assayed for total antioxidant capacity. Values are presented as mM Trolox equivalents, an antioxidant standard. Values are mean ± SD.

We found that significant antioxidant activity was present in all components of the cell culture media (FBS 12.12 [SD 0.1669] mM Trolox equivalents, L929-conditioned media 6.966 [SD 0.074], and RPMI 1.789 [0.093]). Additionally, BMDM supernatant under all experimental conditions demonstrated robust TAC, greater than expected by simple addition of TAC of each of the constituents (Fig 2). However, we found no significant difference in TAC after treatment with LPS or hyperoxia. We thus sought to investigate transcriptional regulation of antioxidant genes within BMDM, but cautiously interpreted our findings, suspecting that the antioxidant activity present in the media would prevent the BMDM from “experiencing” oxidative stress.

### LPS induces a pro-inflammatory phenotype

For our first gene expression studies, we examined the effect of LPS stimulation on inflammation-related gene expression in BMDM cultured under 21% oxygen (room air). Because FBS has antioxidant activity, we exchanged standard cell culture media containing 10% FBS with serum with reduced 5% FBS; we found a further reduction in FBS resulted in significant cell death which precluded further experiments.

**Fig 3:**
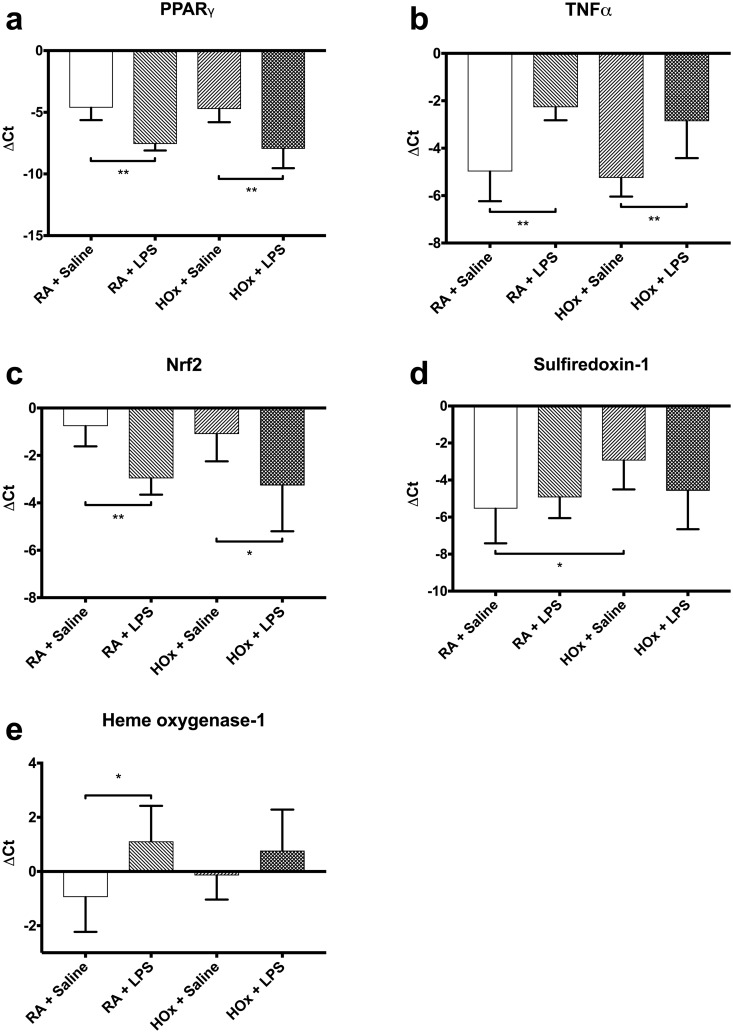
ΔCt values for genes of interest in BMDM. Cells treated with LPS or saline control, and placed in room air (RA) or hyperoxia (HOx). ΔCt values represent difference in threshold cycle between gene of interest and GAPDH. Values are mean ± SD. * p<0.05, ** p<0.01.

**Fig 4:**
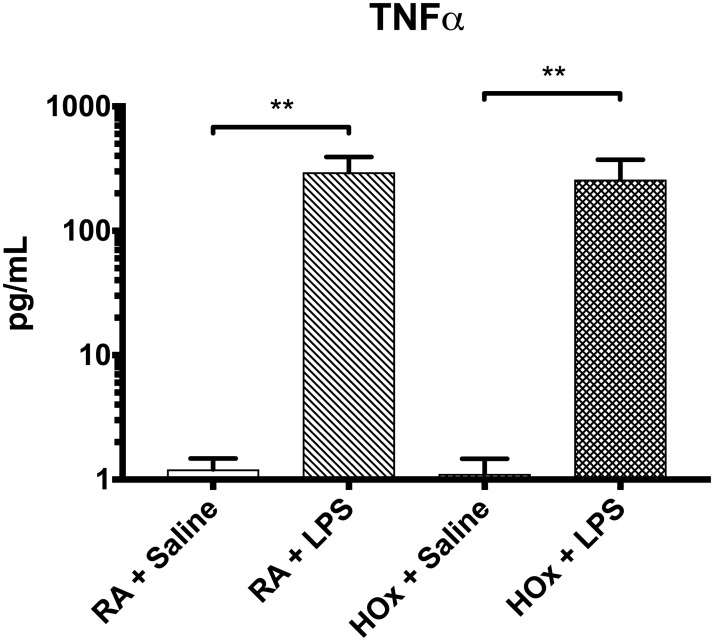
TNFα values in BMDM supernatant. Cells were treated with LPS or saline control, and placed in room air (RA) or hyperoxia (HOx). TNFα levels of the cell culture supernatant were measured via Luminex ELISA. Values are mean ± SD. ** p<0.01.

In room air, LPS treatment induced a pro-inflammatory phenotype (Fig 3a and 3b), as evidenced by decreased PPARγ expression (ΔCt from -4.632 [SD 1.006] to -7.568 [SD 0.529], p = 0.002) and increased TNFα (ΔCt from -4.983 [SD 1.253] to -2.27 [SD 0.553], p = 0.007), relative to room air-exposed saline controls. This same decrease in PPARγ (ΔCt from -4.738 [SD 1.066] to -7.961 [SD 1.568], p = 0.002) and increase in TNFα (ΔCt from-5.251 [SD 0.790] to -2.861 [SD 1.560], p = 0.008), relative to saline controls, was observed in hyperoxia-exposed BMDM. As expected, LPS treatment also increased TNFα protein levels in room air and hyperoxia (Fig 4) (1.21 [SD 0.3] to 295.3 [SD 96.6] pg/mL in room air, and 1.11 [SD 0.4] to 257.9 [SD 114.4] in hyperoxia).

### Pro-inflammatory stimulation diminishes antioxidant gene expression

**Fig 5:**
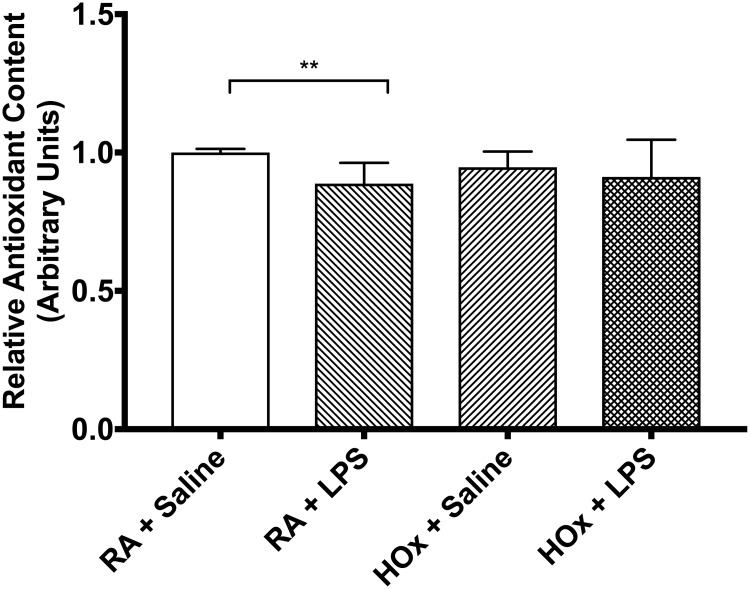
Total antioxidant capacity of BMDM homogenate. Relative total antioxidant capacity per protein content, normalized to room air (RA) samples. Values are mean ± SD. ** p<0.01.

We then studied the effect of LPS stimulation on antioxidant-related gene expression (Fig 3c–3e). In room air, LPS treatment suppressed Nrf2 gene expression (ΔCt from -0.763 [SD 0.856] to -2.968 [SD 0.686], p = 0.002) and increased Hmox1 expression (ΔCt from -0.945 [SD 1.284] to 1.113 [SD 1.310], p = 0.015) relative to saline controls, but did not significantly impact Srxn1 gene expression (ΔCt from -5.545 [SD 1.870] to -4.934 [SD 1.122], p>0.999). LPS stimulation also significantly decreased total antioxidant capacity by 11.2% (p = 0.005) (Fig 5).

In hyperoxia, LPS treatment suppressed Nrf2 gene expression (ΔCt from -1.079 [SD 1.153] to -3.269 [SD 1.932], p = 0.046) relative to saline controls, but had no effect on expression of Srxn1 (ΔCt from -2.947 [SD 1.560] to -4.582 [SD 2.074], p = 0.345) or Hmox1 (ΔCt from -0.147 [SD 0.891] to 0.772 [SD 1.512], p = 0.391). In hyperoxia, LPS stimulation did not significantly decrease total antioxidant capacity (94.7 vs 91.2%, p>0.999) (Fig 5).

### Hyperoxia increases antioxidant gene expression

Our next experiment examined the effect of hyperoxia on gene expression in the absence of LPS (Fig 3c–3e). Hyperoxia treatment increased Srxn1 expression (ΔCt from-5.545 [SD 1.870] to -2.947 [SD 1.560], p = 0.022) relative to room air controls, with no significant effect on expression of PPARγ (ΔCt from -4.632 [SD 1.006 to -4.738 [SD 1.066]), TNFα (ΔCt from -4.983 [SD 1.253] to -5.251 [SD 0.790]), Nrf2 (ΔCt from -0.763 [SD 0.856] to -1.097 [SD 1.153]), or Hmox1 (ΔCt from -0.9452 [SD 1.284] to -0.1467 [SD 0.891]) (p>0.999 for all). Hyperoxia treatment did not significantly alter total antioxidant capacity (100 vs 94.7%, p = 0.313) (Fig 5).

### LPS and hyperoxia treatment is not synergistic

Finally, we examined the effect of hyperoxia on gene expression in the presence of LPS (Fig 3c–3e). Hyperoxia treatment had no significant effect on expression of PPARγ (ΔCt from -7.568 [SD 0.529] to -7.961 [SD 1.568]), TNFα (ΔCt from -2.27 [SD 0.553] to -2.861 [SD 1.560]), Nrf2 (ΔCt from -2.968 [SD 0.686] to -3.269 [SD 1.932]), Srxn1 (ΔCt from -4.934 [SD 1.122] to -4.582 [SD 2.074]), or Hmox1 (ΔCt from 1.113 [SD 1.310] to 0.772 [SD 1.512]) (p>0.999 for all). Hyperoxia treatment did not significantly alter total antioxidant capacity (88.8 vs 91.2%, p>0.999) (Fig 5).

## Discussion

BPD is a chronic lung disease experienced by prematurely born neonates, whose underlying pathophysiology includes inflammatory and oxidative stressors. In this study, we sought to investigate the molecular mechanisms by which these stressors communicate and potentiate damage, and have shown that the anti-inflammatory and anti-oxidant pathways interact in an *in vitro* macrophage model of BPD pathogenesis. We demonstrate how stimulation with LPS increases inflammation and reduces anti-oxidant gene expression, and how stimulation with hyperoxia increases anti-oxidant gene expression but only in the absence of LPS. These findings highlight the complex, multifactorial origins of BPD, contributing to our understanding of BPD pathogenesis.

Because inflammation and oxidative stress are important in BPD pathogenesis, we chose to study PPARγ, TNFα, Nrf2, Srxn1, and Hmox1. PPARγ has typically been studied in metabolic and cardiovascular diseases, but is also an important anti-inflammatory regulator due to its down-regulation of NFκB [14,15]. Additionally, addition of the PPARγ agonist rosiglitazone prevents hyperoxia-induced alveolar simplification [16–18], pulmonary inflammation [17], airway hyperreactivity [16], and pulmonary hypertension [18]. TNFα is a down-stream target of PPARγ and is the canonical pro-inflammatory cytokine implicated in a variety of diseases, including lung injury in preterm neonates [19].

Nrf2 is the master transcriptional regulator of the anti-oxidant response and is known to be important in lung disease, as mice deficient in Nrf2 experience higher mortality, have increased pulmonary edema and inflammation [20,21], and develop pathologic changes similar to those found in BPD [8,22–29]. Furthermore, PPARγ and Nrf2 act in concert to protect the lung against hyperoxia-induced injury [8].

We have demonstrated that LPS produces a pro-inflammatory phenotype, as reflected by decreased expression of anti-inflammatory PPARγ and increased expression of pro-inflammatory TNFα. While this effect under room air (normoxia) is well reported in the literature [14,30,31], this study is the first *in vitro* study to our knowledge that has characterized the pro-inflammatory effect of LPS in BMDM under hyperoxic conditions. We have also shown that pro-inflammatory stimulation reduces the gene expression of anti-oxidant Nrf2 in both normoxic and hyperoxic environments and reduces total antioxidant capacity in normoxia, suggesting that inflammation reduces anti-oxidant defense.

Surprisingly, co-treatment with both LPS and hyperoxia did not result in a synergistic change in gene expression for most of our genes of interest. While hyperoxia increased gene expression of Srxn1, this was only observed in saline-treated BMDM; hyperoxia did not produce any significant increase in BMDM treated with LPS, and hyperoxia did not significantly alter total antioxidant capacity. We speculate, but have not confirmed, that this may be due to other antioxidant molecules which are not regulated by Nrf2, such as catalase [32]. Conversely, LPS treatment increased gene expression of Hmox1, but only in room air; LPS treatment under hyperoxia did not produce a significant change in gene expression. We speculate that the molecular basis for our observations may involve the Akt/PKB and NFκB pathways. It is known that LPS binding TLR4 activates Akt, which regulates antioxidant gene expression via Nrf2 [33], and that reactive oxygen species activate NFκB [34,35]. Our results may suggest a link between these important cellular signaling pathways.

One strength of our study is that we treated BMDM with LPS prior to hyperoxia exposure. Other studies of BPD have focused on one or the other, but in the clinical setting, antenatal infection, such as maternal chorioamnionitis, often precipitates preterm birth, and prematurely born neonates experience a relatively hyperoxic environment. This occurs due to the transition from relatively hypoxic intra-uterine life to the ambient extra-uterine environment, and is exacerbated by the administration of therapeutic supplemental oxygen [36]. Therefore, to model this clinical scenario, we treated BMDM with a pro-inflammatory stimulus two hours prior to placing them in a hyperoxic environment.

Another strength of our study is the investigation of cell culture conditions on studies of oxidative stress. We show that the BMDM media constituents have significant anti-oxidant activity, which may have protected the cells from hyperoxia and obscured significant changes in gene expression. We found that BMDM cultured in standard 10% FBS showed few changes in gene expression, but complete removal of FBS or L929-conditioned media resulted in cell death which precluded RT-PCR experiments. FBS has been shown to include the antioxidant enzyme superoxide dismutase [37] and may contain important antioxidant precursors, such as pyruvate, selenium [38], or alpha-tocopherol [39]. For example, a study in rodent astroglioma cells reported an effect of the serum content of preceding cell culture media on resistance to hydrogen peroxide, which persisted after changing to serum-free media [39]. Therefore, we believe the results from BMDM treated with reduced FBS more accurately reflect the cell’s response to oxidative stress, rather than the response of the cell culture system.

Our study does have important limitations. While BMDM are important in the response to inflammation and oxidative stress, they are not identical to alveolar macrophages, the lung-resident macrophage. However, obtaining alveolar macrophages in sufficient quantities is difficult [11]. We also considered using peritoneal macrophages, but obtaining sufficient quantities would require recruitment such as via thioglycollate injection, which would induce a different phenotype than un-activated macrophages [40]. We chose BMDM because they represent a well-established and well-studied model of the human immune system [10,41], and are easily obtained in abundant quantities. While other cell types are undoubtedly important in the pathogenesis of BPD, macrophages produce many signaling molecules implicated in the pathogenesis of BPD [6] and lie at the intersection of inflammation and oxidative stress [42–48]. An alternative approach of analyzing whole lung homogenates might be informative, but the heterogeneous cell population contained therein would make establishing the role of immune cells difficult to ascertain.

Second, our study is limited in its investigation of predominantly gene expression. Although protein level expression is important, for this study we sought to develop a rapid *in vitro* model in which to test possible therapeutics for BPD, and chose transcriptional regulation as the most accessible end point. We considered evaluating protein expression levels, but as PPARγ and Nrf2 are transcription factors, assaying nuclear translocation would be more technically challenging, while assaying total cell levels would be less informative. We did, however, evaluate levels of TNFα produced by BMDM in response to LPS and hyperoxia to validate our results.

Finally, we chose to study the effect of severe hyperoxia, 95% oxygen, which is not routinely used in current clinical practice. We did evaluate moderate hyperoxia as an oxidative stressor, and did not find any qualitative difference in cell survival or appearance between BMDM kept in room air, moderate hyperoxia, or severe hyperoxia. Therefore, to elicit the greatest response to an oxidative stressor, we used 95% hyperoxia in our model system.

In conclusion, we have established an *in vitro*, primary cell line-based model system with which we can investigate gene expression in response to inflammatory and oxidative stresses as relevant to BPD. We have demonstrated an interaction between the anti-inflammatory and anti-oxidant pathways with respect to gene expression, which we speculate may underlie altered host susceptibility to a “double hit” of infection and oxygen therapy. Future investigations into BPD pathogenesis should incorporate such dual-stimulation to more closely model the clinical scenario. Therapies that decrease the pro-inflammatory response or augment the anti-oxidant response may represent beneficial interventions for BPD.

## Supporting information

S1 Table Data. Includes total antioxidant capacity values and ΔCt from Taqman RT-PCR (normalized to GAPDH) and TNFα values from Luminex.
(XLSX)

## References

[1] Stoll BJ, Hansen NI, Bell EF, Shankaran S, Laptook AR, Walsh MC, et al Neonatal outcomes of extremely preterm infants from the NICHD Neonatal Research Network. Pediatrics. 2010;126: 443–56. doi: 10.1542/peds.2009-2959 20732945 10.1542/peds.2009-2959 PMC2982806

[2] Wilson-Costello D, Walsh MC, Langer JC, Guillet R, Laptook AR, Stoll BJ, et al Impact of postnatal corticosteroid use on neurodevelopment at 18 to 22 months’ adjusted age: effects of dose, timing, and risk of bronchopulmonary dysplasia in extremely low birth weight infants. Pediatrics. 2009;123: e430–7. doi: 10.1542/peds.2008-1928 19204058 10.1542/peds.2008-1928 PMC2846831

[3] Johnson TJ, Patel AL, Jegier BJ, Engstrom JL, Meier PP. Cost of morbidities in very low birth weight infants. J Pediatr. 2013;162: 243–49.e1. doi: 10.1016/j.jpeds.2012.07.013 22910099 10.1016/j.jpeds.2012.07.013 PMC3584449

[4] Keller RL, Ballard RA. Bronchopulmonary Dysplasia In: Gleason CA, Devaskar SU, editors. Avery’s Diseases of the Newborn. 9th ed Philadelphia, PA: Elsevier; 2012 pp. 658–671.

[5] Bancalari E, Walsh MC. Bronchopulmonary Dysplasia in the Neonate In: Martin RJ, Fanaroff A, Walsh MC, editors. Fanaroff and Martin’s Neonatal-Perinatal Medicine. 10th ed Philadelphia, PA: Elsevier; 2015 pp. 1157–1169.

[6] Bose CL, Dammann CEL, Laughon MM. Bronchopulmonary dysplasia and inflammatory biomarkers in the premature neonate. Arch Dis Child Fetal Neonatal Ed. 2008;93: F455–61. doi: 10.1136/adc.2007.121327 18676410 10.1136/adc.2007.121327

[7] Poggi C, Dani C. Antioxidant strategies and respiratory disease of the preterm newborn: an update. Oxid Med Cell Longev. 2014;2014: 721043 doi: 10.1155/2014/721043 24803984 10.1155/2014/721043 PMC3996983

[8] Cho H-Y, Gladwell W, Wang X, Chorley B, Bell D, Reddy SP, et al Nrf2-regulated PPAR{gamma} expression is critical to protection against acute lung injury in mice. Am J Respir Crit Care Med. 2010;182: 170–82. doi: 10.1164/rccm.200907-1047OC 20224069 10.1164/rccm.200907-1047OC PMC2913232

[9] Copple IM. The Keap1-Nrf2 cell defense pathway—a promising therapeutic target? Adv Pharmacol. 2012;63: 43–79. doi: 10.1016/B978-0-12-398339-8.00002-1 22776639 10.1016/B978-0-12-398339-8.00002-1

[10] Davis BK. Isolation, culture, and functional evaluation of bone marrow-derived macrophages. Methods Mol Biol. 2013;1031: 27–35. doi: 10.1007/978-1-62703-481-4_3 23824883 10.1007/978-1-62703-481-4_3

[11] Zhang X, Goncalves R, Mosser DM. The isolation and characterization of murine macrophages. Curr Protoc Immunol. 2008;Chapter 14: Unit 14.1 doi: 10.1002/0471142735.im1401s83 19016445 10.1002/0471142735.im1401s83 PMC2834554

[12] Zhang X, Edwards JP, Mosser DM. The expression of exogenous genes in macrophages: obstacles and opportunities. Methods Mol Biol. 2009;531: 123–43. doi: 10.1007/978-1-59745-396-7_9 19347315 10.1007/978-1-59745-396-7_9 PMC2821576

[13] Livak KJ, Schmittgen TD. Analysis of relative gene expression data using real-time quantitative PCR and the 2(-Delta Delta C(T)) Method. Methods. 2001;25: 402–8. doi: 10.1006/meth.2001.1262 11846609 10.1006/meth.2001.1262

[14] Ricote M, Huang JT, Welch JS, Glass CK. The peroxisome proliferator-activated receptor(PPARgamma) as a regulator of monocyte/macrophage function. J Leukoc Biol. 1999;66: 733–9. 10577502 10.1002/jlb.66.5.733

[15] Chawla A. Control of macrophage activation and function by PPARs. Circ Res. 2010;106: 1559–69. doi: 10.1161/CIRCRESAHA.110.216523 20508200 10.1161/CIRCRESAHA.110.216523 PMC2897247

[16] Takeda K, Okamoto M, de Langhe S, Dill E, Armstrong M, Reisdorf N, et al Peroxisome proliferator-activated receptor-g agonist treatment increases septation and angiogenesis and decreases airway hyperresponsiveness in a model of experimental neonatal chronic lung disease. Anat Rec (Hoboken). 2009;292: 1045–61. doi: 10.1002/ar.20921 19484746 10.1002/ar.20921 PMC2873208

[17] Dasgupta C, Sakurai R, Wang Y, Guo P, Ambalavanan N, Torday JS, et al Hyperoxia-induced neonatal rat lung injury involves activation of TGF-{beta} and Wnt signaling and is protected by rosiglitazone. Am J Physiol Lung Cell Mol Physiol. 2009;296: L1031–41. doi: 10.1152/ajplung.90392.2008 19304912 10.1152/ajplung.90392.2008 PMC3286237

[18] Lee HJ, Lee YJ, Choi CW, Lee J-A, Kim E-K, Kim H-S, et al Rosiglitazone, a peroxisome proliferator-activated receptor-gamma agonist, restores alveolar and pulmonary vascular development in a rat model of bronchopulmonary dysplasia. Yonsei Med J. Korea (South); 2014;55: 99–106. doi: 10.3349/ymj.2014.55.1.99 24339293 10.3349/ymj.2014.55.1.99 PMC3874901

[19] Iliodromiti Z, Zygouris D, Sifakis S, Pappa KI, Tsikouras P, Salakos N, et al Acute lung injury in preterm fetuses and neonates: mechanisms and molecular pathways. J Matern Fetal Neonatal Med. 2013;26: 1696–704. doi: 10.3109/14767058.2013.798284 23611524 10.3109/14767058.2013.798284

[20] Cho H-Y, van Houten B, Wang X, Miller-DeGraff L, Fostel J, Gladwell W, et al Targeted deletion of nrf2 impairs lung development and oxidant injury in neonatal mice. Antioxid Redox Signal. 2012;17: 1066–82. doi: 10.1089/ars.2011.4288 22400915 10.1089/ars.2011.4288 PMC3423869

[21] Reddy NM, Kleeberger SR, Kensler TW, Yamamoto M, Hassoun PM, Reddy SP. Disruption of Nrf2 impairs the resolution of hyperoxia-induced acute lung injury and inflammation in mice. J Immunol. 2009;182: 7264–71. doi: 10.4049/jimmunol.0804248 19454723 10.4049/jimmunol.0804248 PMC2820248

[22] McGrath-Morrow S, Lauer T, Yee M, Neptune E, Podowski M, Thimmulappa RK, et al Nrf2 increases survival and attenuates alveolar growth inhibition in neonatal mice exposed to hyperoxia. Am J Physiol Lung Cell Mol Physiol. 2009;296: L565–73. doi: 10.1152/ajplung.90487.2008 19151108 10.1152/ajplung.90487.2008 PMC2670765

[23] Huang H-C, Nguyen T, Pickett CB. Phosphorylation of Nrf2 at Ser-40 by protein kinase C regulates antioxidant response element-mediated transcription. J Biol Chem. 2002;277: 42769–74. doi: 10.1074/jbc.M206911200 12198130 10.1074/jbc.M206911200

[24] Bloom DA, Jaiswal AK. Phosphorylation of Nrf2 at Ser40 by protein kinase C in response to antioxidants leads to the release of Nrf2 from INrf2, but is not required for Nrf2 stabilization/accumulation in the nucleus and transcriptional activation of antioxidant response element. J Biol Chem. 2003;278: 44675–82. doi: 10.1074/jbc.M307633200 12947090 10.1074/jbc.M307633200

[25] Apopa PL, He X, Ma Q. Phosphorylation of Nrf2 in the transcription activation domain by casein kinase 2 (CK2) is critical for the nuclear translocation and transcription activation function of Nrf2 in IMR-32 neuroblastoma cells. J Biochem Mol Toxicol. 2008;22: 63–76. doi: 10.1002/jbt.20212 18273910 10.1002/jbt.20212

[26] Chen H-H, Chen Y-T, Huang Y-W, Tsai H-J, Kuo C-C. 4-Ketopinoresinol, a novel naturally occurring ARE activator, induces the Nrf2/HO-1 axis and protects against oxidative stress-induced cell injury via activation of PI3K/AKT signaling. Free Radic Biol Med. 2012;52: 1054–66. doi: 10.1016/j.freeradbiomed.2011.12.012 22245092 10.1016/j.freeradbiomed.2011.12.012

[27] Leoncini E, Malaguti M, Angeloni C, Motori E, Fabbri D, Hrelia S. Cruciferous vegetable phytochemical sulforaphane affects phase II enzyme expression and activity in rat cardiomyocytes through modulation of Akt signaling pathway. J Food Sci. 2011;76: H175–81. doi: 10.1111/j.1750-3841.2011.02311.x 22417554 10.1111/j.1750-3841.2011.02311.x

[28] Lee J-W, Bae CJ, Choi Y-J, Kim S-I, Kwon Y-S, Lee HJ, et al 3,4,5-Trihydroxycinnamic acid inhibits lipopolysaccharide (LPS)-induced inflammation by Nrf2 activation in vitro and improves survival of mice in LPS-induced endotoxemia model in vivo. Mol Cell Biochem. 2014;390: 143–53. doi: 10.1007/s11010-014-1965-y 24474616 10.1007/s11010-014-1965-y

[29] Liu Z, Wang J, Huang E, Gao S, Li H, Lu J, et al Tanshinone IIA suppresses cholesterol accumulation in human macrophages: role of heme oxygenase-1. J Lipid Res. 2014;55: 201–13. doi: 10.1194/jlr.M040394 24302760 10.1194/jlr.M040394 PMC3886659

[30] Ricote M, Valledor AF, Glass CK. Decoding transcriptional programs regulated by PPARs and LXRs in the macrophage: effects on lipid homeostasis, inflammation, and atherosclerosis. Arterioscler Thromb Vasc Biol. 2004;24: 230–9. doi: 10.1161/01.ATV.0000103951.67680.B1 14592855 10.1161/01.ATV.0000103951.67680.B1

[31] Welch JS, Ricote M, Akiyama TE, Gonzalez FJ, Glass CK. PPARgamma and PPARdelta negatively regulate specific subsets of lipopolysaccharide and IFN-gamma target genes in macrophages. Proc Natl Acad Sci U S A. 2003;100: 6712–7. doi: 10.1073/pnas.1031789100 12740443 10.1073/pnas.1031789100 PMC164512

[32] Glorieux C, Zamocky M, Sandoval JM, Verrax J, Calderon PB. Regulation of catalase expression in healthy and cancerous cells. Free Radic Biol Med. 2015;87: 84–97. doi: 10.1016/j.freeradbiomed.2015.06.017 26117330 10.1016/j.freeradbiomed.2015.06.017

[33] Reddy NM, Potteti HR, Vegiraju S, Chen H-J, Tamatam CM, Reddy SP. PI3K-AKT Signaling via Nrf2 Protects against Hyperoxia-Induced Acute Lung Injury, but Promotes Inflammation Post-Injury Independent of Nrf2 in Mice. PLoS One. 2015;10: e0129676 doi: 10.1371/journal.pone.0129676 26075390 10.1371/journal.pone.0129676 PMC4467869

[34] Morgan MJ, Liu Z. Crosstalk of reactive oxygen species and NF-κB signaling. Cell Res. 2011;21: 103–15. doi: 10.1038/cr.2010.178 21187859 10.1038/cr.2010.178 PMC3193400

[35] Gloire G, Legrand-Poels S, Piette J. NF-kappaB activation by reactive oxygen species: fifteen years later. Biochem Pharmacol. 2006;72: 1493–505. doi: 10.1016/j.bcp.2006.04.011 16723122 10.1016/j.bcp.2006.04.011

[36] Kapadia VS, Chalak LF, Sparks JE, Allen JR, Savani RC, Wyckoff MH. Resuscitation of preterm neonates with limited versus high oxygen strategy. Pediatrics. 2013;132: e1488–96. doi: 10.1542/peds.2013-0978 24218465 10.1542/peds.2013-0978 PMC3838529

[37] Baret A, Emerit I. Variation of superoxide dismutase levels in fetal calf serum. Mutat Res. 1983;121: 293–7. 6621591 10.1016/0165-7992(83)90217-8

[38] Sandström BE, Carlsson J, Marklund SL. Variations among cultured cells in glutathione peroxidase activity in response to selenite supplementation. Biochim Biophys Acta. 1987;929: 148–53. 3593778 10.1016/0167-4889(87)90170-4

[39] Brenner S, Gülden M, Maser E, Seibert H. Lasting effect of preceding culture conditions on the susceptibility of C6 cells to peroxide-induced oxidative stress. Toxicol In Vitro. 2010;24: 2090–6. doi: 10.1016/j.tiv.2010.06.005 20558276 10.1016/j.tiv.2010.06.005

[40] Schneider M. Collecting Resident or Thioglycollate-Elicited Peritoneal Macrophages. Methods Mol Biol. 2013;1031: 37–40. doi: 10.1007/978-1-62703-481-4_4 23824884 10.1007/978-1-62703-481-4_4

[41] Manzanero S. Generation of mouse bone marrow-derived macrophages. Methods Mol Biol. 2012;844: 177–81. doi: 10.1007/978-1-61779-527-5_12 22262442 10.1007/978-1-61779-527-5_12

[42] Bast A, Erttmann SF, Walther R, Steinmetz I. Influence of iNOS and COX on peroxiredoxin gene expression in primary macrophages. Free Radic Biol Med. 2010;49: 1881–91. doi: 10.1016/j.freeradbiomed.2010.09.015 20869433 10.1016/j.freeradbiomed.2010.09.015

[43] Abbas K, Breton J, Planson A-G, Bouton C, Bignon J, Seguin C, et al Nitric oxide activates an Nrf2/sulfiredoxin antioxidant pathway in macrophages. Free Radic Biol Med. 2011;51: 107–114. doi: 10.1016/j.freeradbiomed.2011.03.039 21466852 10.1016/j.freeradbiomed.2011.03.039

[44] Diet A, Abbas K, Bouton C, Guillon B, Tomasello F, Fourquet S, et al Regulation of peroxiredoxins by nitric oxide in immunostimulated macrophages. J Biol Chem. 2007;282: 36199–205. doi: 10.1074/jbc.M706420200 17921138 10.1074/jbc.M706420200

[45] Kim H, Jung Y, Shin BS, Kim H, Song H, Bae SH, et al Redox regulation of lipopolysaccharide-mediated sulfiredoxin induction, which depends on both AP-1 and Nrf2. J Biol Chem. 2010;285: 34419–28. doi: 10.1074/jbc.M110.126839 20826812 10.1074/jbc.M110.126839 PMC2966056

[46] Tae Lim Y, Sup Song D, Joon Won T, Lee Y-J, Yoo J-S, Eun Hyung K, et al Peroxiredoxin-1, a possible target in modulating inflammatory cytokine production in macrophage like cell line RAW264.7. Microbiol Immunol. 2012;56: 411–9. doi: 10.1111/j.1348-0421.2012.00453.x 22486405 10.1111/j.1348-0421.2012.00453.x

[47] Billiet L, Furman C, Larigauderie G, Copin C, Brand K, Fruchart J-C, et al Extracellular human thioredoxin-1 inhibits lipopolysaccharide-induced interleukin-1beta expression in human monocyte-derived macrophages. J Biol Chem. 2005;280: 40310–8. doi: 10.1074/jbc.M503644200 16207716 10.1074/jbc.M503644200

[48] Carlson BA, Yoo M-H, Conrad M, Gladyshev VN, Hatfield DL, Park JM. Protein kinase-regulated expression and immune function of thioredoxin reductase 1 in mouse macrophages. Mol Immunol. 2011;49: 311–6. doi: 10.1016/j.molimm.2011.09.001 21943784 10.1016/j.molimm.2011.09.001 PMC3205301

